# Lipid-enriched reduced nutrient culture medium improves bovine blastocyst formation

**DOI:** 10.1530/RAF-23-0057

**Published:** 2023-12-14

**Authors:** Rolando Pasquariello, Mingxiang Zhang, Jason R Herrick, Alison F Ermisch, John Becker, William B Schoolcraft, Jennifer P Barfield, Ye Yuan, Rebeca L Krisher

**Affiliations:** 1 Colorado Center for Reproductive Medicine, Lone Tree, Colorado, USA; 2 Colorado State University, Fort Collins, Colorado, USA; 3 Omaha’s Henry Doorly Zoo and Aquarium, Omaha, Nebraska, USA; 4 Genus PLC, DeForest, Wisconsin, USA

**Keywords:** embryo development, lipid, metabolism, gene expression

## Abstract

The refinement of embryo culture media is essential in improving embryo viability and *in vitro* production efficiency. Our previous work demonstrated that the nutrients (carbohydrates, amino acids, and vitamins) in traditional culture media far exceed the need for an embryo and producing developmentally competent embryos in a reduced nutrient environment is feasible. Here, we aim to evaluate the impact of exogenous lipid and l-carnitine supplementation on bovine blastocyst development and refine our RN condition further. Zygotes were cultured in the control medium (100% nutrients) and reduced nutrient media containing 6.25% of the standard nutrient concentrations supplemented with l-carnitine and lipid-free or lipid-rich BSA. Increased blastocyst development was observed in the reduced nutrient lipid-rich medium compared to the other two groups. However, in both reduced nutrient conditions, blastocyst cell numbers were lower than those obtained in the control condition. We then examined the expression level of 18 transcripts correlated with lipid metabolism, glucose metabolism, redox balance, and embryo quality, along with mitochondrial DNA copy numbers, ATP productions, and lipid profile. The results indicated that lipid metabolism, embryo quality, and redox enzyme-related genes were upregulated while the glucose-related gene was downregulated in embryos derived from reduced nutrient lipid-rich condition. Finally, we identified that the lipid-rich BSA has enriched linoleic, stearic, oleic, palmitic, and alpha-linoleic fatty acids, a lipid profile that may contribute to the increased lipid metabolism and improved blastocyst development of the bovine embryos under the reduced nutrient condition.

**Lay summary:**

Assisted conception is a vital technique for cattle breeding. Embryos can be produced by IVF and grow in a special substance known as embryo culture medium. Previous studies suggested that culture medium with a high level of nutrients may have a negative impact on embryo growth. Here, we developed a special culture medium with very low levels of carbohydrates, amino acids, and vitamins, which contained more lipids and a special compound, to make it easier for lipids to move into the cells. The cattle embryos in this culture medium used more lipids and less glucose, and develop much better than ones in the culture medium with high levels of nutrients. Our work provides a unique model to study embryo metabolism and to help improve culture medium.

## Introduction

*In vitro* embryo production (IVP) is a reliable and cost-effective technique increasingly used in cattle breeding. In the last decade, the number of bovine blastocysts produced *in vitro* transferred to a recipient has significantly increased worldwide (Viana 2021). However, improvement of pregnancy rates upon transfer of IVP embryos is still a major concern in cattle farming, with significant financial implications (Ferre *et al.* 2020, Hansen 2020). This might be correlated with reduced oocyte quality after *in vitro* maturation, suboptimal *in vitro* culture (IVC) media that resulted in poor embryo quality, cryopreservation of the embryos, pregnancy losses due to poor uterine environment or transfer techniques, etc. (Ferre *et al.* 2020, Hansen 2020). Technological advances have allowed for the study of metabolite use in individual embryos *in vitro*, indicating that mouse, bovine, human, and feline embryos utilize less than 20% of the metabolites supplied in culture medium (Krisher *et al.* 2015, Herrick *et al.* 2020). In mouse, it has been possible to produce embryos *in vitro* using a reduced nutrient medium (Ermisch *et al.* 2020). However, elevated concentrations of pyruvate and lactate in the first step medium and essential amino acids (EAA) and glutamine in the second step medium were needed to support embryo implantation and development after transferring these embryos to a recipient (Ermisch *et al.* 2020). We recently reported that the percentage of bovine blastocyst formation was not affected until nutrient provision was decreased to 6.25% of standard medium concentrations (Herrick *et al.* 2020). These results open a new frontier in medium formulation for *in vitro* embryo culture in cattle, suggesting embryos might not need such a nutrient-rich environment for successful development.

Fatty acids are essential molecules that support cellular membrane structure and promote inter- and intra- cellular signaling (Albanese & Dainiak 2003), as well as being a potent source for the production of ATP via fatty acid beta-oxidation (Schulz 2008). Embryos metabolize fatty acids during pre-implantation development in several species, including humans (Haggarty *et al.* 2006), mouse (Hillman & Flynn 1980) and cattle (Sutton-McDowall *et al.* 2012). In cattle, fatty acid metabolism has been correlated with supplementation of l-carnitine to the culture medium. Particularly, addition of l-carnitine has been beneficial for lipid metabolism and mitochondrial function (Takahashi *et al.* 2013), allowing improved embryo development (Sutton-McDowall *et al.* 2012) and increased lipid metabolism (Takahashi *et al.* 2013, Held-Hoelker *et al.* 2017). l-Carnitine is needed for the transport of fatty acids into mitochondria where beta-oxidation occurs (Chankitisakul *et al.* 2013). Another function of l-carnitine for embryo development is through its antioxidant action to the mitochondria that was described previously (Wu *et al.* 2011, Truong *et al.* 2016, Jiang *et al.* 2019). However, compared to carbohydrates and amino acids, little is known about the effect of fatty acid supplementation during IVC on bovine embryo development. Previous work in our laboratory has demonstrated that bovine embryos become increasingly dependent on fatty acid oxidation when cultured with reduced concentrations of carbohydrates, amino acids, and vitamins (Herrick *et al.* 2020). Importantly, the culture medium used in that study contained a preparation of BSA with very low levels of fatty acids (essentially fatty acid free), so the primary source of fatty acids available to the embryos would have been intracellular lipids. The medium also did not include l-carnitine, an essential cofactor for entry of fatty acids into the mitochondria that has been shown to stimulate lipid metabolism and embryo development (Sutton-McDowall *et al.* 2012, Takahashi *et al.* 2013).

We hypothesized that the development of bovine embryos cultured in a reduced nutrient medium would be improved if the embryos are provided with l-carnitine and additional extracellular fatty acids. The objective of this study was to compare the development of embryos cultured in our standard medium with a full complement of carbohydrates, amino acids, and vitamins and minimal extracellular lipids (lipid-free BSA) with that of embryos cultured in a reduced nutrient medium with l-carnitine and the absence or presence of extracellular fatty acids. To better understand the relationship of metabolic regulation and embryo development, we also examined expression of metabolic genes, along with relative mitochondrial DNA (mtDNA) copy number, ATP production, and lipid content of individual blastocysts. Understanding the molecular mechanisms related to blastocyst development together with improved formulation of IVC medium will help improve IVP technologies for agricultural and biomedical purposes.

## Materials and methods

Unless specified otherwise, all reagents were purchased from Sigma-Aldrich. The gas concentrations used for *in vitro* maturation (IVM) and *in vitro* fertilization were 7.5% CO_2_ and atmospheric O_2_, and 7.5% CO_2_ and 6.5% O_2_ for IVC. These gas concentrations are increased to compensate for the elevation of our laboratory (~1830 m above sea level) and are approximately equal to 6.0% CO_2_ and 5% O_2_ at sea level (media pH 7.2–7.3) (Herrick *et al.* 2016*a*).

### Quantitative analysis of fatty acid content of albumins

Samples of lipid-free (FAF-BSA) and lipid-rich BSA (FrV–BSA) were dissolved in MilliQ water at 100 mg/mL and analyzed in triplicate over two runs using gas chromatography–mass spectrometry (GC-MS) to determine differences in fatty acid content. To this end, fatty acids bound to albumin were extracted using organic solvent. Liquid samples (350 μL) were first acidified with 20 μL of concentrated HCl, then added with 2 mL of methanol containing 10 μg of C19:0 free acid as internal standard, and 4 mL of chloroform. After a brief vortexing, 1.15 mL of water were added, followed by 10 min vigorous mixing. Then the mixture was centrifuged at 3750 g for 10 min. Chloroform extract in the lower phase was recovered and the solvent was removed under nitrogen. To the dried sample, 200 μL of 3 M methanolic HCl were added and the sample was incubated at 60°C for 1 h. After derivatization, the sample was cooled to ambient temperature, added with 300 μL of hexane and 900 μL of water, vortexed for 1 min, and centrifuged at 1000 ***g*** for 1 min. The hexane layer on the top was recovered and concentrated to various volumes to be injected to GC-MS. Samples (1 μL) were injected onto a Thermo Trace 1310 GC coupled to an ISQ-LT MS. The injector was held at 250°C and a 30:1 split ratio. MS transfer line and source were both held at 250°C. FAME (fatty acid methyl ester) separation was achieved on a 30 m DB-WAX UI column (J&W, 0.25 mm ID, 0.25 μm film thickness). The oven temperature was held at 200°C for 1 min, ramped at 10°C/min to 250°C and held for 1 min. Mass detector was operated under full scan mode (50–650 m/z, 5 scans/s) and electron impact ionization. Calibration curves were prepared by a series of dilutions of authentic standards of fatty acids which were derivatized as described earlier.

### Oocyte *in vitro* maturation

Ovaries were collected at an abattoir by a commercial supplier (BPO Parts LLC, CO, USA) and transported to the laboratory in warmed 0.9% saline. Bovine cumulus–oocyte complexes (COC) were isolated from antral follicles (2–6 mm) within 2.5 h of ovary collection and washed two to three times in 3-(*N*-morpholino)-propanesulfonic acid (MOPS)-buffered medium. *In vitro* maturation was carried out for 22–24 h in groups of five to seven COC in 50 µL drops of a defined maturation medium containing 50 ng/mL recombinant murine EGF, 0.1 IU/mL recombinant human FSH (Follistim, Merck & Co., Inc.), 0.125 mg/mL recombinant human hyaluronan (Novozymes, Bagsvaerd, Denmark), and 2.5 mg/mL recombinant human albumin (AlbIX, Novozymes) under oil.

### *In vitro* fertilization and embryo culture

After IVM, COC were washed using a commercial, serum-free fertilization medium (BO-IVF; IVF Bioscience, Falmouth, UK) and transferred to 45 μL drops of BO-IVF (10–12 COC/drop) under oil. Cryopreserved spermatozoa from a single bull were thawed and processed by density gradient centrifugation (45%:90%, PureSperm, Nidacon, Mölndal, Sweden), followed by two washes in a MOPS-buffered medium. Spermatozoa were diluted with BO-IVF medium and added to drops containing COCs for a final concentration of 1 × 10^6^ spermatozoa/mL. Gametes were co-incubated 18–20 h.

Presumptive zygotes were removed from fertilization drops and denuded of remaining cumulus cells and loosely bound spermatozoa by shaking on a vortex mixer for 2.5 min. After three washes in MOPS-buffered medium, groups of 10 zygotes were randomly allocated to 20 μL drops of serum-free, bovine Optimized Embryo Culture Medium 1(bOEC1) (Herrick *et al.* 2020). On day 3 (72 h in bOEC1, 96 h post insemination), cleavage to at least the two-cell stage was evaluated and embryos with more than four cells were washed and transferred to 20 μL drops of fresh, bovine optimized embryo culture medium 2 designed for compaction and blastocyst formation (Herrick *et al.* 2020). For the final 96 h of culture, embryos were cultured in groups of 5 in 20 μL drops under oil.

Concentrations of salts (NaCl, KCl, KH_2_PO_4_, CaCl_2_ · 2H_2_O, MgSO_4_ · 7H_2_O, and NaHCO_3_), antibiotics (gentamicin, 25 µg/mL), macromolecules (hyaluronan, 0.125 mg/mL), and growth factors (insulin, transferrin, and selenium, ITS) were kept the same in all treatments to maintain consistent osmolarity and pH. Nutrients (glucose/fructose, citrate, lactate, pyruvate, amino acids, vitamins, and EDTA) were diluted to 6.25% of control (reduced nutrient, RN). The control medium was supplemented with 8 mg/mL lipid-free BSA (Herrick *et al.* 2016*b*). The RN media were supplemented with 5 mM l-carnitine (Sutton-McDowall *et al.* 2012) and 8 mg/mL of lipid-free or lipid-rich BSA.

### Determination of blastocyst cell number and allocation

**Figure 1:**
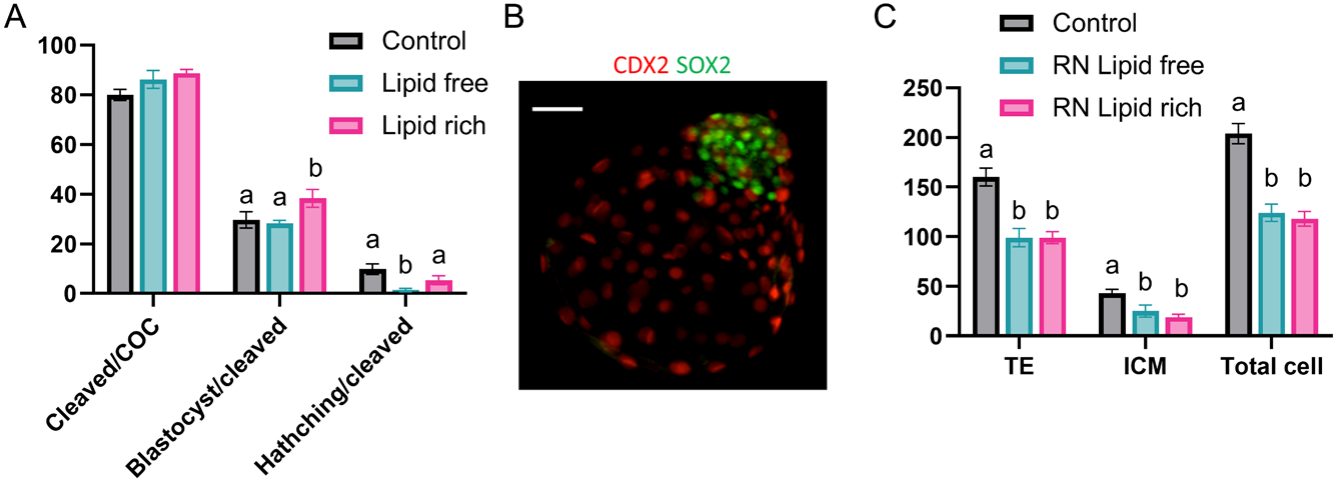
Effects of culture environment on embryo development and blastocyst cell numbers. (A) Embryo development in control medium and reduced nutrient (RN) media supplemented lipid-free and lipid-rich BSA (control, *n* = 587; RN lipid free, *n* = 573; RN lipid rich, *n* = 585, the experiments were replicated seven times). The percentage of cleaved embryos was calculated of the total number of cumulus–complex oocytes matured and fertilized (cleaved/COC); the percentage of blastocysts (blastocyst/cleaved) and of hatching/fully hatched blastocysts (hatching/cleaved) of cleaved embryos were calculated. (B) A representative image of a bovine blastocyst stained by CDX2 (red) and SOX2 (green) to assess blastocyst cell numbers. Scale bar, 20 µm. (C) The number of cells in the trophectoderm (TE) and inner cell mass (ICM), and the total number of cells were determined in bovine blastocysts from control (*n* = 39), RN lipid-free (*n* = 6) and RN lipid-rich (*n* = 20) media. Data are reported as mean ± s.e.m. Different superscripts indicate a significant difference between treatments (*P* < 0.05).

Expanded, hatching and fully hatched blastocysts were fixed for 20 min in 4% paraformaldehyde (Electron Microscopy Sciences, Hatfield, PA, USA) and then stored in PBS with 0.5% BSA (MP Biomedicals) until staining. Blastocysts were washed three times in PBS with 0.1% polyvinylpyrrolidone (PVP) and 0.1% Triton X-100 (TX100) and then permeabilized in PBS with 1.0% TX100 (30 min). After blocking (2 h) in PBS with 0.1% TX100, 0.1 M glycine, 0.5% BSA, and 10% (v/v) horse serum, blastocysts were incubated with primary antibodies (18 to 24 h, 4°C) for SRY-box 2 (SOX2, Biogenex, Fremont, CA, rabbit monoclonal, anti-human; AN579) and caudal type homeobox 2 (CDX2, Biogenex, mouse monoclonal, anti-human; MU392A) (Bakhtari & Ross 2014, Herrick *et al.* 2016*b*) to detect inner cell mass (ICM) and trophectoderm (TE) cells, respectively. Following three washes in PBS with 0.1% PVP and 0.1% TX100, blastocysts were incubated (1 h) with secondary antibodies (Alexa Fluor 488 donkey anti-rabbit IgG (A-2126, SOX2) and Alexa Fluor 555 goat anti-mouse IgG (A-21424, CDX2; Invitrogen, ThermoFisher Scientific). Blastocysts were washed three times and mounted on a glass slide in ProLong Gold Antifade reagent containing DAPI (Life Technologies, ThermoFisher Scientific). Cells were visualized using a fluorescent microscope (Olympus BX52) and counted using the manual count function of MetaMorph software. Cells positive for SOX2 were considered ICM cells and cells positive for CDX2 were considered TE cells (Fig 1B). The total number of cells in the blastocyst was calculated as the sum of SOX2- and CDX2-positive cells.

### Gene expression analysis

Gene expression analysis was carried out by real-time quantitative PCR (RT-qPCR) for the following genes (Table 1): fatty acid metabolism: *ACADL*, *ACSL3, ECHS1*, *MT-CO2*, *CPT1B*, *PPARGC1A*; glucose metabolism: *HK1*, *LDHA*, *PDHA1*, *PDK1,TIGAR*, *TALDO1*; embryo quality: *PLAC8*, *POU5F1*, *PTGS2;* redox balance: *GLRX2*, *TXNRD1*, *SOD1*. A total of four biological replicates (pools of eight hatching and fully hatched blastocysts) for each treatment (control, lipid-free and lipid-rich RN media) were used for after snap freezing in liquid nitrogen in 10 µL PBS + 0.01 % PVP. RNA extraction was performed using the PicoPure RNA Isolation Kit (ThermoFisher Scientific) with on-column DNase treatment (Qiagen). Complementary DNA (cDNA) was synthesized using iScript™ cDNA Synthesis Kit (Bio-Rad) following the manufacturer’s protocol. The cDNA samples were diluted 1:3 using RNase-free water and stored at −20°C until qPCR was run. For each biological sample, PCR was performed in duplicate using 12.5 µL Power SYBR™ Green PCR Master Mix (Applied Biosystems), 2.5 µL 10 µM primer mix and 5 µL 1:3 diluted cDNA sample. The qPCR program was as follows: 50° C for 2 min for the first cycle, 95°C for 2 min for the second cycle, followed by 40 cycles of amplification step at 95°C for 10 s and 59°C for 1 min. A melting curve was analyzed for each experiment to assess the specificity of primer amplification. Relative gene expression was calculated using the 2^−ΔΔCt^ method (Pfaffl 2001). Normalization of Ct values was obtained using expression of *GAPDH*.

**Table 1:** Primer sequences used for gene expression analysis.

Name ID	Accession number ID	Forward primer (5′ -> 3′)	Reverse primer (5′ -> 3′)
*ACADL*	NM_001076936.1	CCCGTGTCCAGACAATCTATG	GATGTGGGCAGATGTCTACTG
*ACSL3*	NM_001205468.2	TTGGCTTTCCTACGAAGATG	CCCTGGTCTCACAGAAGATG
*CPT1B*	NM_001034349.2	CCAAGAACATCTCCGGAAACA	ATTCCCTCCAGCCCTACTT
*ECHS1*	NM_001025206.2	TCAGAGTGAAGGCCTTGTTG	CAAGGCCTAATGTGACCTGAA
*HK1*	NM_001012668.2	CTGCTTGACAAAGCCATCAA	CGTCGTAGCCACAGGTCAT
*GAPDH*	NM_001034034.2	TCATCATCTCTGCACCTTCTG	ATGCCAAAGTGGTCATGGA
*GLRX2*	NM_001040523.2	CCCGCACTAAGACCATGTA	CTGCATTTCCCAAAGATGA
*LDHA*	NM_174099.2	CAGATTGCAACCACTTCCA	GCAAGTTGCTTGTTGTTTCC
*MT-CO2*	NC_006853.1	CCAAGATGCAACATCACCAATC	TGGGTCAGCTTTGTCGTTAG
*PLAC8*	NM_001025325.2	TGAACGAATGCTGCCTATGG	CAGGCAATCCTTGCAAATGG
*POU5F1*	NM_174580.3	GCCAAGCTCCTAAAGCAGAA	TTGAAACTGAGCTGCAAAGC
*TXNRD1*	NM_174625.5	AGGCAGCCAAATATGACAAG	CGTAGGGCTTGACCTAACAA
*PPARGC1A*	NM_177945.3	TTGCCCAGATCTTCCTGAAC	CACTTGAGTCCACCCAGAAA
*PDK1*	NM_001205957.1	GTCACCAGCCAGAATGTTCA	TCCGATGAGATAGGCTTCCT
*PTGS2*	NM_174445.2	AAGATCTCCTTCCTGCGAAA	ATCAGGCACAGGAGGAAGAG
*PDHA1*	NM_001101046.2	AGAGTGCTGGTGGCATCTC	GAACGGTCTGCATCATCCT
*SOD1*	NM_174615.2	CTTCGAGGCAAAGGGAGATAC	CTTGTGTATTGTCTCCAAACTGATG
*TIGAR*	NM_001076370.1	GAGTGCCCAGCATTCACA	CATTCTGACCGGCTTCTTTC
*TALDO*	NM_001035283.2	GCGCCTCATTGAGCTGTA	CTGGGCGAAGGAGAAGAG

### Mitochondrial DNA copy number of individual blastocysts

The quantification of relative mtDNA copy number per cell was carried out using individual blastocysts collected from control, RN lipid-free and RN lipid-rich treatments as previously described (Wu *et al.* 2015). Briefly, blastocysts were washed in 1× PBS without calcium and magnesium with 0.01% PVP, collected individually and stored in 10 μL PBS + 0.01% PVP at −80°C. DNA extraction was carried out using the QIAamp DNA micro kit (Qiagen) according to the manufacturer’s protocol with RNA carrier (1 μg/μL; supplied with the kit) added to each sample. The DNA samples were analyzed using RT-qPCR to calculate mtDNA copy number relative to the amount of nuclear DNA. Primer sequences for amplification of mtDNA and nuclear DNA (i.e. *GAPDH*) are detailed in Table 2. RT-qPCR of mitochondrial DNA and nuclear DNA was performed simultaneously for each sample in triplicate using SYBR green PCR master mix (Applied Biosystems) and QuantStudio 5 real-time machine (Applied Biosystems). The program of amplification was as follows: 50°C for 2 min for the first cycle; 95° C for 2 min for the second cycle; 95°C per 15 s, 60°C for 30 s and 72°C per 1 min for 40 cycles. The Ct value for *GAPDH* was subtracted from that for bovine mtDNA region to give the ΔCt value. mtDNA copy number per nuclear genome (two *GAPDH* gene copies) was calculated as 2 × 2^ΔCt^. DNA samples of each category were compared on the same RT-qPCR plate in order to produce comparable results.

**Table 2:** Primer sequences used for relative mitochondrial DNA copy number assay of individual bovine blastocysts.

Name ID	Accession number ID	Forward primer (5′ -> 3′)	Reverse primer (5′ -> 3′)
mtDNA region	AY526085.1	GGGCTACATTCTCTACACCAAG	GTGCTTCATGGCCTAATTCAAC
GAPDH	AB098985.1	ATATAGCTGCCTGACCTTTCTG	GGATTGGGAGCAACAGGTATTA

### Lipid profile of individual blastocysts

Individual blastocysts obtained from control and RN lipid-rich treatments were washed several times using PBS + 0.01% PVP before collection using 1 µL of PBS + 0.01% PVP, snap freezing and storage at −80°C. Lipids were extracted from D7 blastocysts using 100% methanol, and the extracts were profiled using a chromatographically coupled time of flight mass spectrometer (LC-MS). Briefly, a lipid extract of each sample was injected onto a Waters Acquity UPLC system in discrete, randomized blocks with a pooled QC injection after every eight sample injections. The sample injections were then separated using a Waters Acquity UPLC CSH Phenyl Hexyl column (1.7 μM, 1.0 × 100 mm), using a gradient made of 2 mM ammonium hydroxide + 0.1% formic acid and acetonitrile + 0.1% formic acid. The column and samples were held at 65°C and 6°C, respectively. The column eluent was infused into a Waters Xevo G2 TOF-MS with an electrospray source in positive mode. The scanning was run using 50–2000 *m*/*z* at 0.2 s module, alternating MS (6 V collision energy) and MSE mode (15–30 V ramp). Calibration was performed using sodium iodide with 1 ppm mass accuracy. The capillary voltage was held at 2200 V, source temp at 150°C, and nitrogen desolvation temperature at 350°C with a flow rate of 800 L/h. Data analysis was run using a non-targeted data acquisition (GC-MS and UPLC-MS). For each sample, raw data files were converted to .cdf format, and a matrix of molecular features as defined by retention time and mass (m/z) was generated using XCMS software in R (Smith *et al.* 2006) for feature detection and alignment. Raw peak areas were quantile normalized. While outlier injections were detected based on total signal and PC1 of principal component analysis (PCA), the mean area of the chromatographic peak was calculated among replicate injections for QC samples. Features were grouped using RAMClustR (Broeckling *et al.* 2014), which groups features into spectra based on co-elution and covariance across the full dataset. Spectra are used to determine the identity of observed compounds in the experiment. Compounds were annotated based on spectral matching to in-house, NISTv14, 1- SToP spectral databases (Broeckling *et al.* 2016).

### ATP quantification

Individual blastocysts obtained from control, lipid-free and lipid-rich RN treatments were collected in 10 µL of PBS + 0.01% PVP and frozen at −80°C. ATP concentrations were determined by the ATP bioluminescent somatic cell assay kit (Sigma Chemical Co.) as previously described with minor modifications (Cukurcam *et al.* 2004, Simsek-Duran *et al.* 2013, Silva *et al.* 2015). Briefly, 1:5 diluted ATP assay mix was added to individual wells in an opaque 96-well plate. In a separate tube, somatic cell ATP-releasing reagent was mixed with each sample or standards, and added to the assay mix. The amount of light emitted was immediately measured using a Synergy 2 plate reader for luminescence (BioTek). ATP concentration was calculated by comparison to a standard curve ranging from 60 fmol to 2 pmol/100 µL and normalized (per cell) using the average total cell number for blastocysts from each treatment.

### Statistical analysis

Statistical analyses were completed using IBM SPSS (IBM). The Student’s *t*-test (two treatments) or the one-way ANOVA (multiple comparisons) test was conducted depending on the number of experimental groups. For the analysis of lipid content, ANOVA was conducted on each compound using the analysis of variance function of R package. For this analysis, *P*-values were adjusted for false positives using the Bonferroni–Hochberg method using p.adjust function of R package. PCA was performed on mean-centered and Pareto variance-scaled data using the R package pcaMethods (Stacklies *et al.* 2007). Results were considered statistically significant when *P* < 0.05. Unless otherwise stated, results are presented as mean ± s.e.m.

## Results

### Determination of fatty acid abundance in lipid-free and lipid-rich BSA

The lipid-rich BSA had a total concentration of 14.3 µg of fatty acids per mg of albumin, whereas the lipid-free BSA had 0.13 µg of fatty acids per mg of albumin (Table 3). There was a higher concentration (µg per mg albumin) of several fatty acids in lipid-rich BSA with respect to lipid-free BSA including linoleic (C18:2, 3.21), stearic (C18:0, 3.07), oleic (C18:1, 2.87), palmitic (C16:0; 2.71), and alpha-linoleic fatty acids (C18:3, 1.83) (Table 3). However, because the overall amount of fatty acid was considerably lower, the total concentration of even the most abundant fatty acid, palmitic acid, in lipid-free BSA, was only 0.04 µg per mg of albumin compared to 2.7 µg palmitic acid per mg of albumin in lipid-rich BSA.

**Table 3:** Fatty acid content (µg per mg albumin) of lipid-free and lipid-rich BSA used in these experiments.

	Fatty acid								
	Total	Palmitic	Stearic	Oleic	Linoleic	Alpha-linolenic	Palmitoleic	Myristic	Margaric
		(C16:0)	(C18:0)	(C18:1)	(C18:2)	(C18:3)	(C17:0)	(C16:1)	(C14:0)
Lipid-free BSA	0.13	0.04	0.03	0.03	0.00	0.00	0.00	0.01	0.01
Lipid-rich BSA	14.33	2.71	3.07	2.87	3.21	1.83	0.31	0.16	0.17

### Effect of culture environment on embryo development

Our objective in this experiment was to investigate the effect of exogenous lipid availability on embryo development in an RN culture environment. Embryo cleavage (≥ 79.9%) was not different between treatments (Fig. 1A). However, blastocyst development (per cleaved embryos) was higher in the RN lipid-rich medium compared to the control and RN lipid-free treatments (Fig. 1A). There was no difference in blastocyst hatching between embryos cultured in RN lipid-rich and control media, while fewer blastocysts hatched in the RN lipid-free treatment group (Fig. 1A). Blastocysts produced in RN lipid-free and RN lipid-rich media had fewer TE, ICM, and total cells than those produced in control medium (TE – control: 160 ± 9.0; RN lipid free: 99.0 ± 5.9; RN lipid rich: 99.0 ± 9.1; ICM – control: 43.0 ± 4.0; lipid free: 19.0 ± 2.9; RN lipid rich: 25.0 ± 6.1; Total – control: 204.0 ± 10.2; RN lipid free: 124.0 ± 8.7; RN lipid rich: 118.0 ± 7.3, Fig. 1B and C)

### Effect of culture environment on blastocyst gene expression

**Figure 2:**
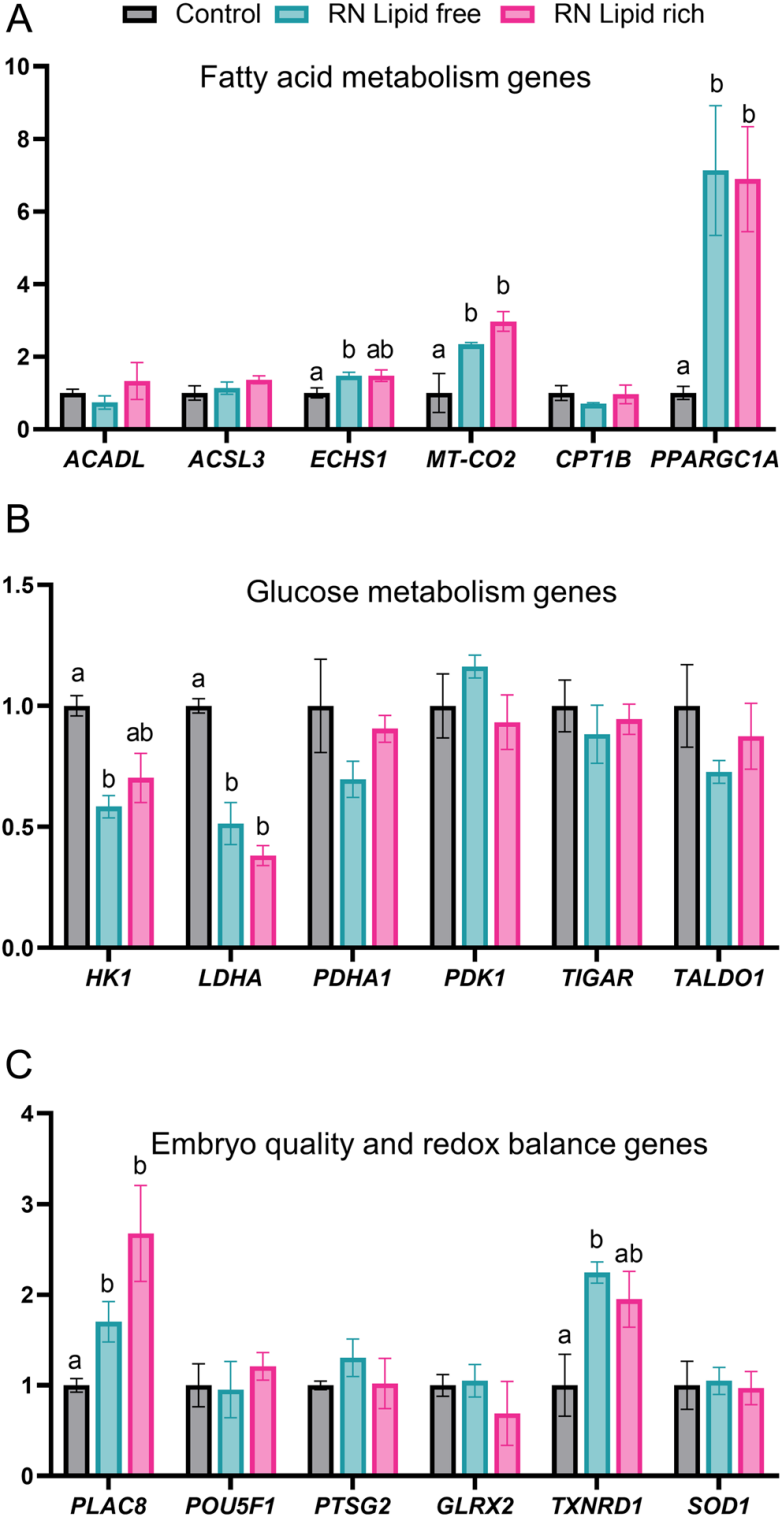
Effect of culture environment on blastocyst gene expression related to fatty acid metabolism (A), glucose metabolism (B) and embryo quality and redox balance (C). The gene expression value was arbitrarily set to 1 for samples from the control medium. Comparisons were made between samples from the control medium with the ones from RN lipid-free and RN lipid-rich media. The experiments were replicated four times. Data are reported as mean ± s.e.m. Different superscripts indicate a significant difference between treatments (*P* < 0.05).

Amongst the fatty acid metabolism genes, *MT-CO2* had higher expression (*P* < 0.05) in both RN lipid-free and lipid-rich media, and *ECHS1* was also upregulated in the RN lipid-free (*P* < 0.05) and RN lipid-rich (*P* = 0.08) media when compared to control medium. *PPARGC1A* followed the same trend and was upregulated (*P* < 0.05) in both RN lipid-free and lipid-rich media (Fig. 2A). The glucose metabolism gene *HK1* had a lower expression in both RN lipid-free (*P* < 0.05) and RN lipid-rich (*P* = 0.05) media, and the glucose metabolism gene *LDHA* was also downregulated (*P* < 0.05) in both RN conditions when compared to the control medium (Fig. 2B). The embryo quality-related gene, *PLAC8*, was upregulated (*P* < 0.05) in both RN lipid-free and RN lipid-rich media, and the redox gene *TXNRD1* was also high in RN lipid-free (*P* < 0.05) and RN lipid-rich (*P* = 0.09) media when compared to the control medium (Fig. 2C).

### Effect of culture environment on blastocyst mtDNA copy number and ATP content

**Figure 3:**
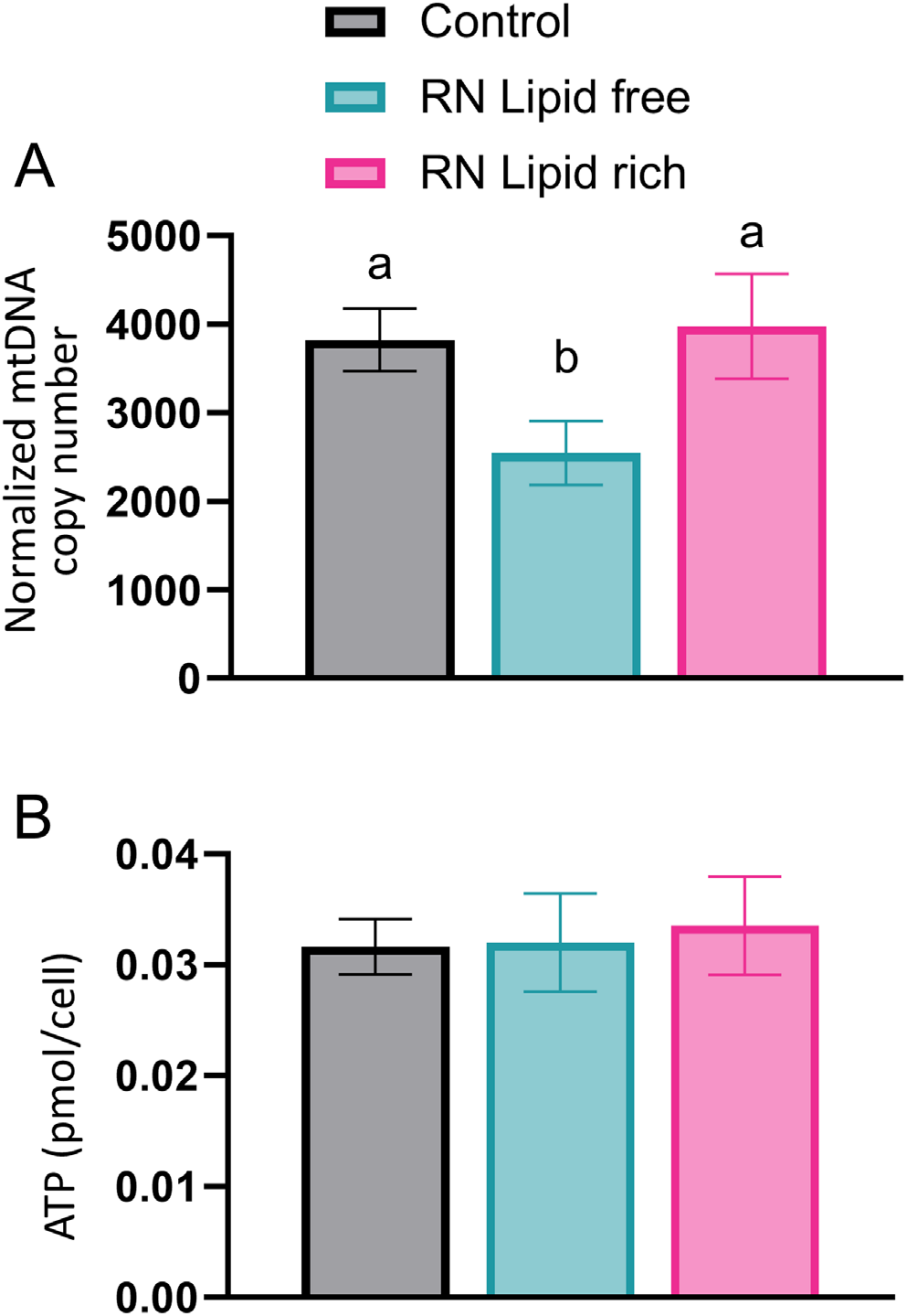
Mitochondrial copy number and ATP quantification of individual bovine blastocysts produced in different culture environments. (A) The quantification of relative mtDNA copy number per cell of individual blastocysts were compared between control (*n* = 19), RN lipid-free (*n* = 18), and RN lipid-rich media (*n* = 19). The experiments were replicated three times. (B) ATP quantification of individual bovine blastocysts were compared between control (*n* = 15), RN lipid-free (*n* = 15), and RN lipid-rich media (*n* = 15). Data are shown as pmoL/cell obtained by dividing the ATP content of individual blastocysts by the average total cell number for that treatment. The experiments were replicated three times. Data are reported as mean ± s.e.m. Different superscripts indicate a significant difference between treatments (*P* < 0.05).

To determine whether the provision of fatty acids in a reducing nutrient environment had an effect on mitochondrial number, the relative mtDNA copy number was assessed. Interestingly, blastocysts produced in RN lipid-free medium had lower relative mtDNA copy number than those produced in control and RN lipid-rich media (Fig. 3A). Blastocyst ATP content normalized per cell was not significantly different between embryos produced in control, RN lipid-free, and RN lipid-rich media (Fig. 3B).

### Effect of culture environment on individual blastocyst lipid profile

**Figure 4:**
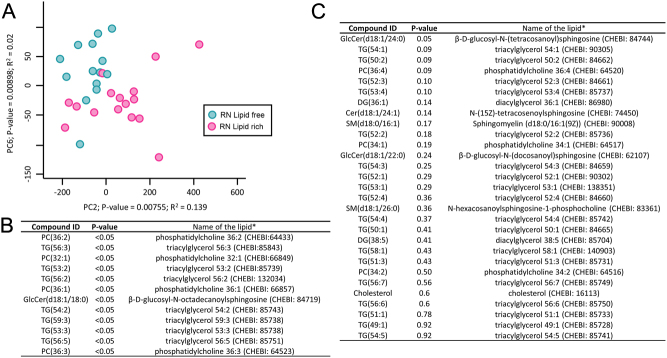
Effect of culture environment on individual blastocyst lipid contents. (A) PCA obtained using lipid content of the blastocysts produced with control (*n* = 14) and RN lipid-rich media (*n* = 15). (B and C) A total of 12 out of 40 tested lipids were different between blastocysts from the control and RN lipid-rich media. Compounds were annotated based on spectral matching to in-house, NISTv14, 1 – SToP spectral databases (Broeckling *et al.* 2016).

The PCA using blastocyst lipid content identified two different clusters that separated the blastocysts obtained from the control and those produced with RN lipid-rich medium (Fig. 4A). A total of 43 lipids were annotated, 12 of which were significantly reduced in blastocysts cultured in RN lipid-rich media compared to control (Fig. 4B).

## Discussion

Our previous work demonstrated that bovine embryos only used a small amount of the nutrients, and the blastocyst formation was not affected until nutrient concentrations were reduced to 6.25% of the nutrients present in control condition (Herrick *et al.* 2020). Such remarkable resilience to reduced concentrations of nutrients may be explained by the ability of bovine embryos to utilize their internal stored lipids as the energy source when other nutrients are scarce. Therefore, we hypothesized that promoting fatty acid oxidation may restore the declined blastocyst formation in the 6.25% RN condition seen in our previous study (Herrick *et al.* 2020). When l-carnitine is included in the 6.25% RN condition, we observed the blastocyst development was restored when compared with the 100% control, and surprisingly, a significantly improved blastocyst development was observed when growing bovine embryos in the 6.25% RN condition with supplementation of lipid-rich BSA (i.e. FrV BSA) and l-carnitine. It is well documented that lipid metabolism, especially the utilization of endogenous lipid, is indispensable for energy production and proper development of bovine embryos (Sutton-McDowall *et al.* 2012, Takahashi *et al.* 2013). The importance of lipid metabolism on bovine embryo development was also demonstrated in our previous work, in which the use of a fatty acid oxidation inhibitor, etoxomir, blocked blastocyst formation and hatching (Herrick *et al.* 2020). However, whether exogenous lipid supplementation would benefit bovine embryo development is debated. Replacing lipid-rich fetal bovine serum (FBS) with low-lipid BSA resulted in improved bovine embryo cryotolerance and quality (Rizos *et al.* 2003, Del Collado *et al.* 2015). It is suspected that the high lipid content in FBS may result in excessive lipid accumulation, thus resulting in compromised embryo quality (Del Collado *et al.* 2015). In a recent mouse study, we also observed compromised blastocyst development when lipid-rich albumins was supplemented in the culture media and compared with the lipid-free albumins (Logsdon *et al.* 2021). However, the variability observed in different albumin products may be not due to the amount of lipids; instead, the different lipid profiles and variations of other contaminants in these albumins may have played bigger roles in embryo development (Logsdon *et al.* 2021). Another important note from our mouse study was that addition of l-carnitine mitigated the blastocyst developmental differences between the lipid-rich and lipid-free albumin (Logsdon *et al.* 2021). l-Carnitine can enhance lipid metabolism and mitochondrial activity during embryo development by facilitating fatty acids transportation into mitochondria, where ATP is generated by fatty acid beta-oxidation (Held-Hoelker *et al.* 2017). In this study, we confirmed the positive effect of exogenous lipid supplementation and the importance of l-carnitine supplementation to facilitate lipid metabolism during embryo development. As shown in earlier studies, when access to other nutrients are readily available, excessive lipid supplementation may be detrimental to bovine embryo development (Rizos *et al.* 2003, Del Collado *et al.* 2015). Therefore, it is important to note that the positive effect we see here may depend on the availability of other nutrients.

In order to elucidate what specific fatty acid profiles are associated with improved embryo development in the lipid-rich RN medium, we performed lipid content analysis on both lipid-free BSA and lipid-rich BSA. These two BSAs have different concentrations of linoleic, stearic, oleic, palmitic, and alpha-linoleic fatty acids (Table 3), which are classified as non-esterified fatty acids (NEFA). Oleic acid was reported as an important energy source and cellular structure in both oocyte and embryo development (Fayezi *et al.* 2018), and high concentration of linoleic and oleic acid in the embryo culture medium resulted in improved bovine embryo recovery from thawing (Karasahin 2019). It is also important to note that the concentrations of the fatty acids used in our study were not as high as those used in other work. Therefore, the beneficial effect of exogenous lipid supplementation on embryo development could be related to the specific fatty acid profiles instead of the quantity, and the fatty acids we identified here may be important candidates to constitute an optimal fatty acid profile for embryo development.

Analysis of genes involved in this study gives us a few hints on how RN environment controls the embryo metabolism and development. HK1 controls phosphorylation of glucose to glucose 6-phosphaste (G6P) and is the gateway enzyme of glucose metabolism that plays a central role in modulating multiple signaling pathways. The activity of hexokinase is closely related to glucose consumption and progressively increased in embryos at morula stage (Houghton *et al.* 1996, Banliat *et al.* 2022). *LDHA* is the gene that is highly expressed in bovine blastocysts (Harvey *et al.* 2007) and controls the conversion of pyruvate to lactate and participate in maintaining redox balance by converting NADH to NAD+. Both *HK1* and *LDHA* were downregulated and genes involved in fatty acid metabolism and mitochondrial oxidation (*ECHS1*, *MT-CO2, PPARGC1A*) were upregulated in blastocysts collected from the RN media. These results indicate that pyruvate was not preferentially converted to lactate in the RN media. Instead, it may be routed to the mitochondria and, along with fatty acids, used for ATP production via oxidative phosphorylation. These results also suggested that embryos from RN environment preferentially relied on fatty acids as the energy source. The shift from glycolysis to fatty acid oxidation could affect the proliferation rate, which might explain the reduced cell number in RN-derived embryos (De Oliveira & Liesa 2020). In addition, such shift in energy source may result in less regeneration of NAD+ and more production of ROS through oxidative phosphorylation, challenging the ability of the embryos in maintaining proper redox balance. This speculation is supported by fact that a higher level of the redox enzyme *TXNRD1* was observed in embryos from RN environment, thus providing a higher antioxidant capacity to counterbalance the elevated ROS level in the embryos. The upregulation of *PLAC8* in the blastocysts from RN conditions is also interesting. Its upregulation in more competent bovine blastocysts have been shown in at least three different studies (Salilew-Wondim *et al.* 2021). PLAC8 promotes autophagy and improves the proliferation of human trophoblast cells (Feng *et al.* 2021), suggesting its potential role in improving bovine embryo implantation via the mechanism of PLAC8-induced autophagy in trophoblast cells.

Blastocysts cultured in RN lipid-rich medium had a higher mtDNA copy number than those from RN lipid-free medium. This is the sign that embryos in RN lipid-rich medium tend to increase the number of mitochondria and utilize more fatty acids as the energy source and shift their metabolism to favor mitochondrial oxidative phosphorylation. Our lipid profile analysis of individual blastocyst supported this notion, as 12 out of 43 annotated lipids containing fatty acids were significantly reduced in blastocysts cultured in RN lipid-rich medium compared to the control. However, the ATP production of individual cells remain similar among all three conditions. The similar ATP production suggested that embryos under different culture conditions have similar cellular energy demands to support their development. However, the embryos in the RN lipid-free condition may experience environmental stress due to nutrient availability. Therefore, mitochondrial replication in these embryos is compromised. As a result, each individual mitochondrial may have to have elevated oxidative phosphorylation activities to meet the ATP demand. It is important to note that the regulation of mitochondrial function, energy production, and metabolism is a highly complex process involving multiple factors and signaling pathways (reviewed in (Harvey 2019)). Additional characterizations, such as mitochondrial activities, redox status, embryo transcriptional and translational activities, may help us better understand the underlying mechanisms.

## Conclusions

In summary, blastocyst development was significantly improved after supplementing fatty acids and l-carnitine to a medium with RN concentrations. The mechanism underlying this phenomenon may be related to increased lipid metabolism in the mitochondria in the RN environment. It is important to note that the benefit of supplementing fatty acid may depend on the availability of other nutrients. The reduced blastocyst cell numbers and increased expression of embryo quality related genes provided conflicting interpretations of the quality of the resulting embryos. In-depth analyses of transcriptome, proteome, and post-transfer embryo viability are needed in the future to fully evaluate the metabolic mechanisms and developmental competence of the embryos produced from the RN environment. The novel RN media may also provide a unique model to study embryo metabolism and facilitate the optimization of culture media. Ultimately, the ability of bovine embryos to develop in an environment with such low concentrations of carbohydrates and amino acids demonstrated the remarkable plasticity of preimplantation embryo development and urged us to reexamine what should be considered as optimal IVC environment.

## Declaration of interest

The authors declare that there is no conflict of interest that could be perceived as prejudicing the impartiality of the study reported.

## Funding

This work was funded by the internal research fund provided by Colorado Center for Reproduction Medicine.

## Author contribution statement

RP and RLK conceived the study; RP, JRH, AFE, JB, and YY performed the experiments; WBS, JPB, and RLK contributed to project administration; RP, MZ, YY analyzed the data; RP, MZ, YY, and RLK wrote the paper.
